# Development of underground detection system using a metal detector and aluminum tag for *Copris ochus* (Coleoptera: Scarabaeidae)

**DOI:** 10.1093/jisesa/ieae067

**Published:** 2024-06-24

**Authors:** Jung-Wook Kho, Young-Joong Kim, Hwang Kim, Sun Hee Hong, Young Su Lee, Jong-Seok Park, Doo-Hyung Lee

**Affiliations:** Department of Life Sciences, Gachon University, Seongnam, South Korea; Division of Restoration Research, National Institute of Ecology, Yeongyang, South Korea; Division of Restoration Research, National Institute of Ecology, Yeongyang, South Korea; School of Plant Science and Landscape Architecture, College of Agriculture and Life Sciences, Hankyong National University, Anseong, South Korea; Gyeonggi-do Agricultural Research and Extension Services, Hwaseong, South Korea; Department of Biological Sciences and Biotechnology, Chungbuk National University, Cheongju, South Korea; Department of Life Sciences, Gachon University, Seongnam, South Korea

**Keywords:** insect tracking, mark-recapture, dispersal, pre-reproductive stage

## Abstract

Tracking of soil-dwelling insects poses greater challenges compared to aboveground-dwelling animals in terrestrial systems. A metal detector system consisting of a commercially available detector and aluminum tags was developed for detecting dung beetle, *Copris ochus* Motschulsky (Coleoptera: Scarabaeidae). First, detection efficacy of the system was evaluated by varying volumes of aluminum tags attached on a plastic model of the insect and also by varying angles. Then, detection efficacy was evaluated by varying depths of aluminum-tagged models under soil in 2 vegetation types. Finally, the effects of tag attachment on *C. ochus* adults were assessed for survivorship, burrowing depth, and horizontal movement. Generally, an increase in tag volume resulted in greater detection distance in semi-field conditions. Maximum detection distance of aluminum tag increased up to 17 cm below soil surface as the tag size (0.5 × 1.0 cm [width × length]) and thickness (16 layers) were maximized, resulting in a tag weight of 31.4 mg, comprising ca. 9% of average weight of *C. ochus* adult. Furthermore, the detection efficacy did not vary among angles except for 90°. In the field, metal detectors successfully detected 5 aluminum-tagged models in 20 × 10 m (W × L) arena within 10 min with detection rates ≥85% for up to depth of 10 cm and 45%–60% at depth of 20 cm. Finally, aluminum tagging did not significantly affect survivorship and behaviors of *C. ochus*. Our study indicates the potential of metal detector system for tracking *C. ochus* under soil.

## Graphical Abstract

**Graphical Abstract:**
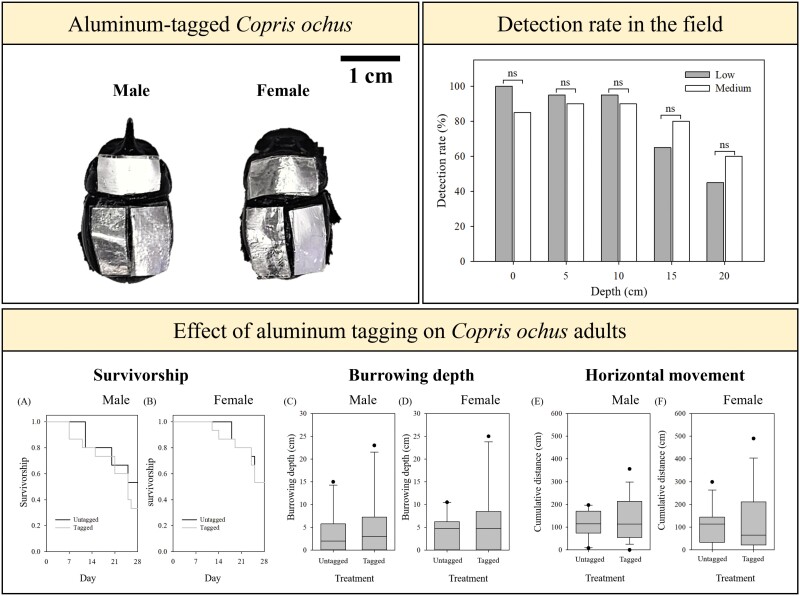

## Introduction

An effective tracking method can help monitor abundance, investigate distribution, and observe behavior of target species (Hagler and Jackson 2001, Riley et al. 2005). However, unlike larger organisms including mammals or birds, developing tracking methods for insects is challenging (Nathan 2001). Insects have smaller body sizes, which limits the sizes and weights of markers and tags to be applied without affecting their physiology or behavior (Boiteau and Colpitts 2001, Kim et al. 2016, Batsleer et al. 2020). For example, whereas a radio collar weighing >35 g can be applied for tracking mammals including deer (Tester et al. 1964) or foxes (Storm 1965), attachment of a radar tag weighing only 3 mg affected the flight pattern of 2 insect species, *Apis mellifera* (Hymenoptera: Apidae) and *Ricania* sp. (Hemiptera: Ricaniidae) (Kim et al. 2016). However, tag size is generally associated with success and efficacy of the detection method; detectable distance of a tracking method and life span of battery-operated radio tags are known to increase with tag size (O’Neal et al. 2004, Wikelski et al. 2007, Kissling et al. 2014).

There has been continuous research effort to develop effective tracking methods for insect species including permanent marking, color marking, and radio tracking (Hagler and Jackson 2001, Kissling et al. 2014, Batsleer et al. 2020). Permanent markings including clipping, puncturing, and mutilation have been used mainly for coleopteran insects due to their hard elytra (Unruh and Chauvin 1993, Butler et al. 2012). Color marking, especially fluorescent marking paired with ultraviolet light source, has been proven effective for investigating abundance and dispersal pattern of multiple insect species including mosquitoes, stinkbugs, and lanternflies (Verhulst et al. 2013, Dickens and Brant 2014, Rice et al. 2015, Kho et al. 2019, Keller et al. 2020, Jung et al. 2022). Radio tracking allows real-time tracking of insect location even without clear sight of the insect unlike color marking. The method can be categorized into 2 types based on the use of internal power source for the tag. Active tags can be detected from 100 to 400 m, but their use is limited depending on the weight and longevity of battery (Negro et al. 2008, Levett and Walls 2011, Drag and Cizek 2018). On the other hand, passive tags due to their lack of battery can be applied to smaller insects, and they are not limited by the longevity of a battery (Osborne et al. 1999, Chapman et al. 2004, O’Neal et al. 2004, Riley et al. 2005).

Although numerous techniques have been developed for tracking terrestrial insects above ground, only few studies have attempted to track insects below the soil. O’Neal et al. (2004) used a commercially available handheld harmonic radar to track soil-dwelling carabid beetles. When tagged with an 8-cm long, 91-mg monopole tag, insects could be detected up to 9 cm below the soil. Another technique to track insects below soil involves a metal detector and a tag. Commercially available metal detectors have been used to track movement and survival of 2 coleopteran larvae, *Cryptocephalus coryli* (Coleoptera: Chrysomelidae) and *Melolontha melolontha* (Coleoptera: Scarabaeidae) up to 7 cm below the soil layer (Piper and Compton 2002, Piper et al. 2014, Bont et al. 2017). In these studies, the use of either portable harmonic radar or metal detector resulted in successful detection of respective coleopteran insects below the soil. However, effects of tagging on physiology and behavior of insects were only partially investigated (Piper and Compton 2002, O’neal et al. 2004) or reported to be significantly adverse (Bont et al. 2017). Furthermore, from both detection methods, the detection distance was within 10 cm below the soil, which may limit their use for detecting insect species that burrows deeper into the soil. For example, *Nicrophorus americanus* (Coleoptera: Silphidae) has been reported to overwinter in burrows that are as deep as 20 cm (Schnell et al. 2008), and dung beetle species belonging to family Geotrupidae may build nest between 8 and 21 cm below soil surface (Huerta et al. 2023).

Dung beetles are important ecosystem engineers in various ecosystems, including tropical forest and coastal dune ecosystems (Cambefort and Hanski 1991, Nichols et al. 2008). They are known to provide multiple ecosystem services including nutrient cycling, bioturbation, seed dispersal, and zoological vector suppression (Bang et al. 2005, Nichols et al. 2008, Arellano et al. 2023). Nevertheless, dung beetles have been decreasing in number and in some areas have become locally extinct due to habitat loss and changes in commercial livestock practice (Lobo 2001, Kim et al. 2021). For a successful conservation and restoration of dung beetles in the ecosystem, their behaviors, including dispersal, foraging, and nesting, in the field need to be understood. For this, it is important to track and locate insects in their natural habitat. However, previous studies on dung beetle behaviors have mostly been conducted above ground by observing captured dung beetles or conducting mark and recapture via color marking (Roslin 2000; Silva and Hernández 2015). That is, information on distribution and behavior was estimated based on individuals lured to the trap instead of a direct tracking of dung beetles.

To track dung beetles in the field, previous detection methods were modified using a commercially available metal detector and metal tags to provide sufficient detection depth without affecting their survivorship and behaviors. With an increase in popularity of metal detectors for recreational purposes, these devices have become more accessible and easier to operate. In general, these metal detectors can detect coin-sized metals up to 10–30 cm under the soil, which varies with conductivity of the metal (MineLab 2022, Wall 2023). In addition, metal detectors are designed adjustable to detect only desired types of metal. Meanwhile, aluminum has a potential for making an effective metal tag, because it is one of the more conductive metals compared to iron or nickel. At the same time, it is easily accessible and affordable than other metals of high conductivity such as gold and silver. Especially, commercially available aluminum foil can be easily manipulated into different shapes, making it a suitable material for metal tags.

The present study aimed to develop a tracking method for detecting dung beetles underground using metal detectors and aluminum tags. For this, detection efficacy of the method was evaluated by varying the size and layer of aluminum tags, and the effectiveness of the metal detector system was tested in potential habitats of dung beetles, varying with vegetation heights and covers. Then, effects of aluminum tag attachment on survivorship, burrowing depth in soil, and horizontal movement of dung beetle were assessed. In this study, a paracoprid, *Copris ochus* Motschulsky (Coleoptera: Scarabaeidae), was selected as model species. This species nests underneath cattle feces for feeding and reproduction, with its brood chambers containing dung balls below the soil surface (Bang et al. 2004). In addition, *C. ochus* has become more important in ecosystem restoration being under consideration as a candidate species for taxon substitution of a locally extinct telecoprid dung beetle species in South Korea (Kim 2020).

## Materials and Methods

### Metal Detector System

In this study, a metal detector system was developed to detect dung beetles under soil, which consisted of a commercially available metal detector (Vanquish 540, Minelab Electronics, South Australia, Australia) and aluminum tags made of aluminum foil (Daehan Wellbeing Foil, Daehan Wellbeing Unbak, Seoul, South Korea). A metal detector generates electromagnetic fields from its coils. When it encounters metal items that conduct electricity, a new magnetic field is generated. In addition, these metal detectors can identify and discriminate metal items detected based on conductivity of the item. In general, metal detectors quantify conductivity and represent it as ID, which can be utilized by operators to specify target material to detect in the field.

### Detection Rates for Various Tag Specifications

To evaluate the performance of the metal detector system, detection depth and associated ID of aluminum tags at varying sizes and thicknesses were assessed. To make aluminum tags of different thicknesses, strips of aluminum foils were folded into varying layers and applied with cyanoacrylate glue at edges to prevent tags from unfolding. The size of *C. ochus* used in this study ranged from 1.2 to 1.6 cm in width and 2.0 to 2.8 cm in length with an average size of ca. 1.5 × 2.5 cm (width × length), and its body weight was 1.06 ± 0.04 g (mean ± SE). Based on this, upper limits of tag size and thickness tested were set at 0.5 × 1.0 cm (W × L) and 16 layers, respectively, so that the tag size would not exceed the area of *C. ochus* elytra and pronotum where tags would be attached, and the tag weight does not exceed 10% of body weight of *C. ochus* (Kim et al. 2016). In total, aluminum tags of 9 different specifications consisting of 3 different sizes (small: 0.25 × 0.5 cm; medium: 0.38 × 0.75 cm; large: 0.5 × 1.0 cm [W × L]) and 3 different thicknesses (4, 8, and 16 layers) were tested (Table 1).

**Table 1.** Tag specifications varying with size (small [0.25 × 0.5 cm], medium [0.38 × 0.75 cm], and large [0.5 × 1.0 cm] [W × L]) and layers (4, 8, and 16) with their maximum detectable distances and ID values of signals read by a metal detector. Three individual tags were attached on a 3D-printed dung beetle model on which detectable distance and signal ID were evaluated under semi-field condition.

Tag size	Tag layer	Individual tag weight (mean ± SE) (mg)	Total tag weight/*C. ochus* body weight (%)^a^	Maximum detectable distance (cm)	Most frequently detected ID^b^
	4	2.1 ± 0.0	0.59	3	1
Small	8	4.3 ± 0.0	1.21	7	1
	16	8.5 ± 0.1	2.41	9	4
	4	4.2 ± 0.0	1.19	6	1
Medium	8	8.1 ± 0.1	2.29	11	2
	16	16.7 ± 0.2	4.73	15	7
	4	7.5 ± 0.1	2.12	9	1
Large	8	16.3 ± 0.3	4.61	13	4
	16	31.4 ± 0.5	8.89	17	9

^a^The percentage of total tag weight to *Copris ochus* adult body weight was calculated based on the average body weight of *C. ochus* (1.06 g) measured in the laboratory.

^b^ID is a parameter evaluated and presented by a metal detector (Vanquish 540—Minelab Electronics, South Australia, Australia) when sensing aluminum-tagged models.

To test the detection efficacy of aluminum tags, we used plastic dummy models of *C. ochus* adults manufactured using 3D printers. The size of the plastic model was set based on average width and length of *C. ochus* adults (1.5 × 2.5 cm [W × L]) to represent both sexes as their sizes did not show significant difference when compared using student’s *t*-test (width: *t* = 1.6, *df *= 58, *P* = 0.12; length: *t* = 1.6, *df *=* *58, *P* = 0.12). Three aluminum tags were attached to a plastic model for each specification. Two tags were attached to each one of the elytra and one tag was attached to the pronotum using cyanoacrylate glue (Supplementary Fig. 1)

To measure the detection depth and ID for aluminum tags of each specification, a plastic box (52 × 35 × 28 cm [width × length × height]) filled with sandy soil was prepared. Another smaller transparent container (5 × 5 × 25 cm [W × L × H]) containing an aluminum-tagged plastic model and the same soil was placed at the center of the plastic box. Each model was buried inside the container at a designated depth ranging from 0 to 23 cm. Then, an operator attempted to detect the buried aluminum-tagged model using a metal detector by hovering and swiping the metal detector above the plastic box. During operation, the metal detector was set to detect signals with ID ranging from 0 to 40 without further adjustment. The operator recorded the success and failure of the detection, which were evaluated based on the auditory signal sent out by the metal detector, as well as the ID read by the metal detector. The experiment was replicated 10 times at each depth. If the metal detector successfully identified the aluminum-tagged model at least once, further tests were carried out by replacing the transparent container with a different container having an aluminum-tagged model buried 1 cm deeper. Linear regression analyses were performed to assess the effect of average tag weight on both the maximum detection depth and the most frequently detected ID (JMP version 12, SAS Institute Inc., NC, United States).

In addition, the effect of orientation of aluminum-tagged model on detection rate was evaluated. The plastic box and container were set up as described above. However, inside the transparent container, aluminum-tagged model was placed in 5 different angles relative to the soil surface: 0°, 45°, 90°, 135°, and 180°, where at 0° the aluminum-tagged model was placed with its dorsal side up while upside down at 180°. Then, detection of aluminum-tagged model at different angles was tested every 5 cm and replicated 10 times for each of the 9 specifications.

### Detection Efficacy in the Field

Detection efficacy of the metal detector systems developed in this study was evaluated in Taean Sinduri Coastal Dune, South Korea (36°84ʹ59.23″N 126°19ʹ71.11″E), where *C. ochus* is currently under consideration as a candidate species for taxon substitution of a locally extinct telecoprid dung beetle species. Prior to assessing detection efficacy of the metal detector system, 2 different environments of the coastal dune were screened to characterize metal items pre-existing in the field, which may affect the detection efficacy of the method. Two sites were selected based on their vegetation height and density. Low vegetation site, where *Imperata cylindrica* (Poales: Poaceae) was the most dominant, consisted of grass under 20 cm in height with plant cover <30%. The medium vegetation site, where *Leymus mollis* (Poales: Poaceae) was the most dominant species, consisted of grass up to 50 cm in height with plant cover up to 70%. In each site, an operator screened a plot of 20 × 10 m (W × L) using a metal detector. When a positive signal was read by the metal detector, its location was marked. Upon completion of screening, the time for completion was recorded, and marked points were revisited to measure the ID and visually identify the type of metals, which were later removed from the sites.

Following the preliminary screening, the screened plot was used as an experimental arena (20 × 10 m [W × L]) in each vegetation site. A recorder hid 5 plastic dummy models of *C. ochus* attached with aluminum tags (large, 16-layered; Table 1) at 5 varying depths (0, 5, 10, 15, and 20 cm) under soil. The recorders, after hiding each dummy model, decided on random direction and distance prior to moving to the next hiding location, to minimize potential biases when hiding the dummy models. Then, an operator searched for aluminum-tagged models in the experimental arena using a metal detector. Based on the previous experiment, where most frequently measured ID for the above tag specification was 9 (Table 1), metal detector was calibrated to detect metals with ID range from 7 to 10. The operator was given a maximum of 10 min to screen the experimental arena during which the operator marked locations where the metal detector sent positive signals. Once a thorough searching was complete, the operator returned to markers and visually detected aluminum-tagged models by digging the soil using a shovel. The operator was given a maximum of 5 min to retrieve the model, and the time to retrieval for each tag was recorded. In each site, 3 operators conducted the experiment for 6–7 times each, resulting in 20 replications in total.

To evaluate potential factors affecting the detection efficacy of metal detector system, first, a generalized linear model was used to determine effects of vegetation site, depth, and their interaction term on detection rate and time to retrieval. In addition, we further compared detection rates between the 2 sites at each depth using Pearson’s chi-squared test. Also, time to retrieval at varying depths was compared within vegetation site using ANOVA (JMP12, SAS). In the analysis, time to retrieval for 0 cm depth was not included, because aluminum-tagged models were visually identified immediately after the metal detector sensed them while marking the location.

### Effect of Aluminum Tagging on Dung Beetles

A series of experiments were conducted to test the potential effect of aluminum tagging on *C. ochus* under laboratory and field conditions. *Copris ochus* adults used in this study were collected from grasslands (<30 cm in height) (33°20ʹ48.20″N 126°40ʹ39.31″E) near horse pastures in Jeju Island in August 2023. We deployed pitfall traps made of a plastic pot (6 × 13 cm [diameter × height]) sealed with a net with a dung ball made from horse feces placed on top as a lure at grasslands. On the next day, *C. ochus* adults captured in the pitfall traps were collected and brought to the laboratory, where they were sexed and maintained in plastic cages (52 × 35 × 28 cm [W × L × H]) filled with sand under 25°C, 30% ± 10%, and 13:11 L:D. Every week, collected *C. ochus* individuals were provided with dung balls made of cattle feces as food sources, which were acquired from grazing cattle in Taean Sinduri Coastal Dune, the target site for dung beetle species recovery (Kim 2020). In addition, dung balls were placed on water-soaked cotton pads in 90-mm petri dishes to keep them moisturized.

Aluminum tags were prepared by folding a strip of aluminum foil into large size and 16 layers (Table 1). Cyanoacrylate glue was applied on edges of tags to prevent unfolding of tags during activities of dung beetles after attachment. Prior to attaching tags onto *C. ochus* adults, the surface of their prothorax and both sides of elytra were gently smoothed by rubbing sandpaper to remove the waxy layer (Lee et al. 2013). Then, aluminum tags were carefully applied onto each surface using cyanoacrylate glue (Supplementary Fig. 1).

The effect of aluminum tagging on survivorship of *C. ochus* was evaluated for 30 untagged and 30 aluminum-tagged adults (sex ratio = 1:1). At the onset of the experiment, adults were individually put into a transparent cylindrical plastic cup (8 × 11 cm [D × H]) filled with sand up to 5 cm, which was sealed with a plastic lid. One dung ball (1 cm diameter) made of cow feces was placed on a 30-mm petri dish with distilled water-soaked cotton pad and provided to each individual every week. These insects were maintained at 25°C, 30% ± 10%, and 13:11 L:D, and their survivorship were checked daily for 28 days, which represent the foraging period of newly emerged *C. ochus* adults prior to settling in their nests for breeding and nidification (Bang et al. 2008). The survivorship of untagged and aluminum-tagged adults was compared within sex using Kaplan–Meier survival analysis (JMP12, SAS).

The effect of aluminum tagging on burrowing depth of *C. ochus* under soil was evaluated for 20 untagged and 20 aluminum-tagged adults (sex ratio = 1:1). An adult was placed inside of a cylindrical plastic bottle (10 × 42 cm [D × H]) filled with sand to the top and provided with a dung ball (1 cm diameter) made of cow feces placed on a 30-mm petri dish with distilled water-soaked cotton pad. The insect was maintained at 25°C, 30% ± 10%, and 13:11 L:D for 24 h. Then, burrowing depths of dung beetles were recorded by carefully digging up the sand until each individual was detected and measuring the distance between the individual and the top. The effect of aluminum tagging, sex, and the interaction between the 2 terms on the burrowing depth of *C. ochus* was analyzed using a generalized linear model (JMP12, SAS).

The effect of aluminum tagging on horizontal movement of *C. ochus* was evaluated for 10 untagged and 10 aluminum-tagged adults (sex ratio = 1:1). Due to a nocturnal/crepuscular nature of *C. ochus*, under dark, a dung beetle was placed on a grassland (<5 cm in height) in Gachon University (37°27ʹ2.44″N 127°7ʹ50.74″E) and given 5 min to acclimate to the environment (Doube 1990). Then, location of each individual was marked every 5 min over 30 min after which the recorder measured the distance between each marker. The experiment was repeated 3 times for each individual and conducted after sunset between 19:30 and 23:00 at 15°C–17°C in late September 2023. The effect of aluminum tagging, sex, and the interaction terms on horizontal walking distance was analyzed using a mixed model, with the identification of insects included as a random factor (JMP12, SAS).

## Results

### Detection Rate for Various Tag Specifications

Overall, the detection distance of aluminum-tagged models increased with increasing tag size and thickness (Table 1). As the tag thickness increased from 4 to 16 layers, the maximum detectable distance was increased by 3-, 2.5-, and 1.9-fold for small, medium, and large tags, respectively. Similarly, the maximum detectable distance increased with an increase in tag size for the 3 tag thicknesses. In addition, the ID measured by the metal detectors also increased with tag size and thickness, although most frequently detected ID was 1 regardless of the tag size when tag thickness was set at 4 layers (Table 1). As the tag thickness increased, the most frequently detected ID increased to 4, 7, and 9 for tag sizes of small, medium, and large, respectively. Finally, linear regression analyses showed that the average weight of aluminum tags was positively correlated with both the maximum detection depth (intercept = 5.10, slope = 0.44, *R*^2^ = 0.83, *P *< 0.001) and the most frequently detected ID (intercept = 0.02, slope = 0.30, *R*^2^ = 0.87, *P *< 0.001).

**Fig. 1.**
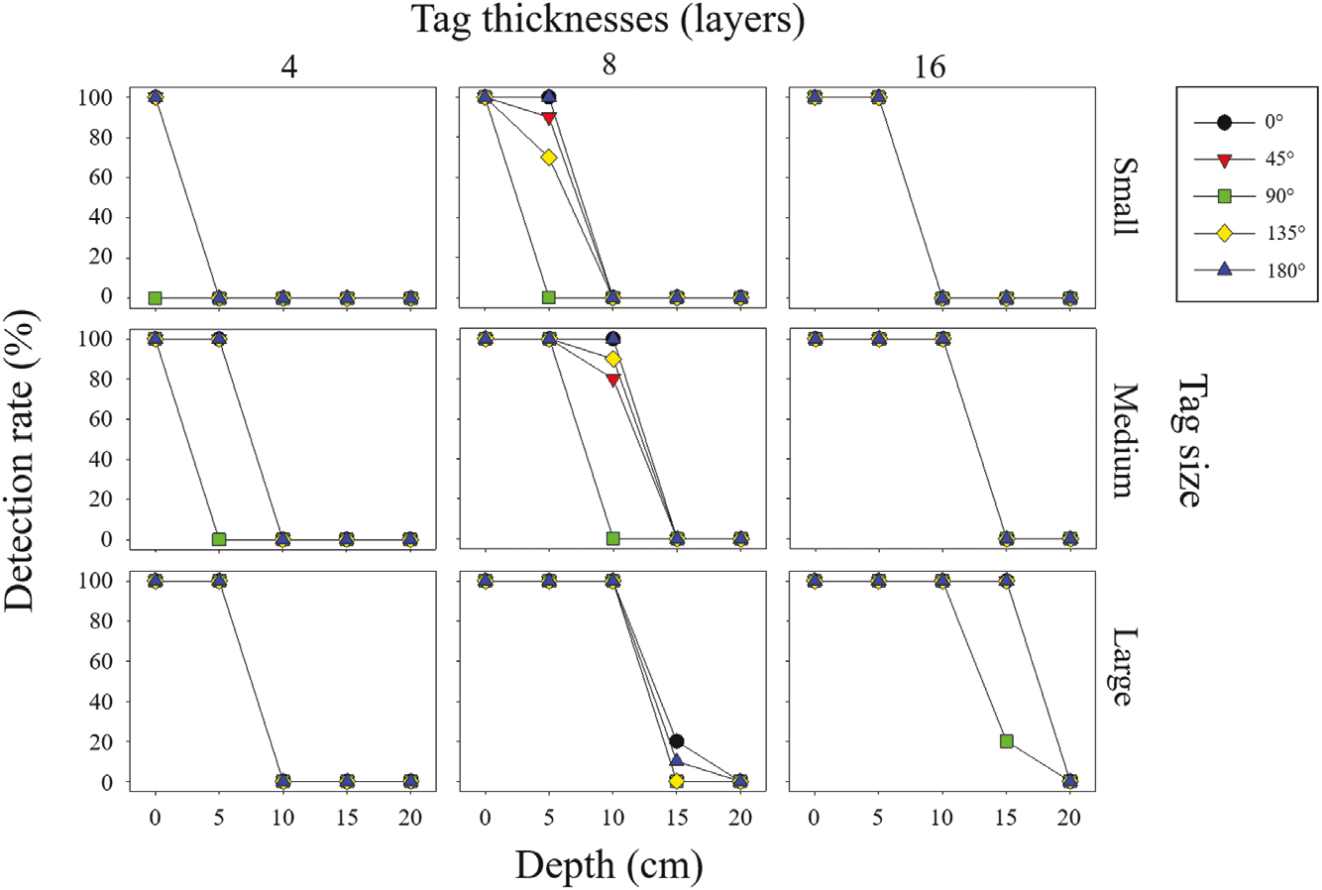
Detection rate of the metal detector system for varying size (small [0.25 × 0.5 cm], medium [0.38 × 0.75 cm], and large [0.5 × 1.0 cm] [W × L]) and thicknesses (4, 8, and 16 layers) of aluminum tags attached on a plastic dung beetle model placed in 5 different orientations respective to the soil surface. Detection rates at 5 angles (0°, 45°, 90°, 135°, and 180°) were evaluated at a depth of every 5 cm from 0 to 20 cm.

Generally, the metal detector system showed similar detection rate across varying orientations. However, at 90°, lower detection rates were distinctly observed (Fig. 1). When tag thickness was 4 layers, 0% detection rate was observed at depths of 0 and 5 cm for small and medium tags at 90°, respectively. Within tag thickness of 8 layers, detection rates were ≥70% at depths of 5 and 10 cm at all orientations for small and medium tags, respectively, except for 90° at which the detection rates were 0%. Finally, at tag thickness of 16 layers, detection rates were maintained the same for all orientations except for large tags, where the detection rate dropped to 20% at an angle of 90° at depth of 15 cm.

### Detection Efficacy in the Field

**Fig. 2.**
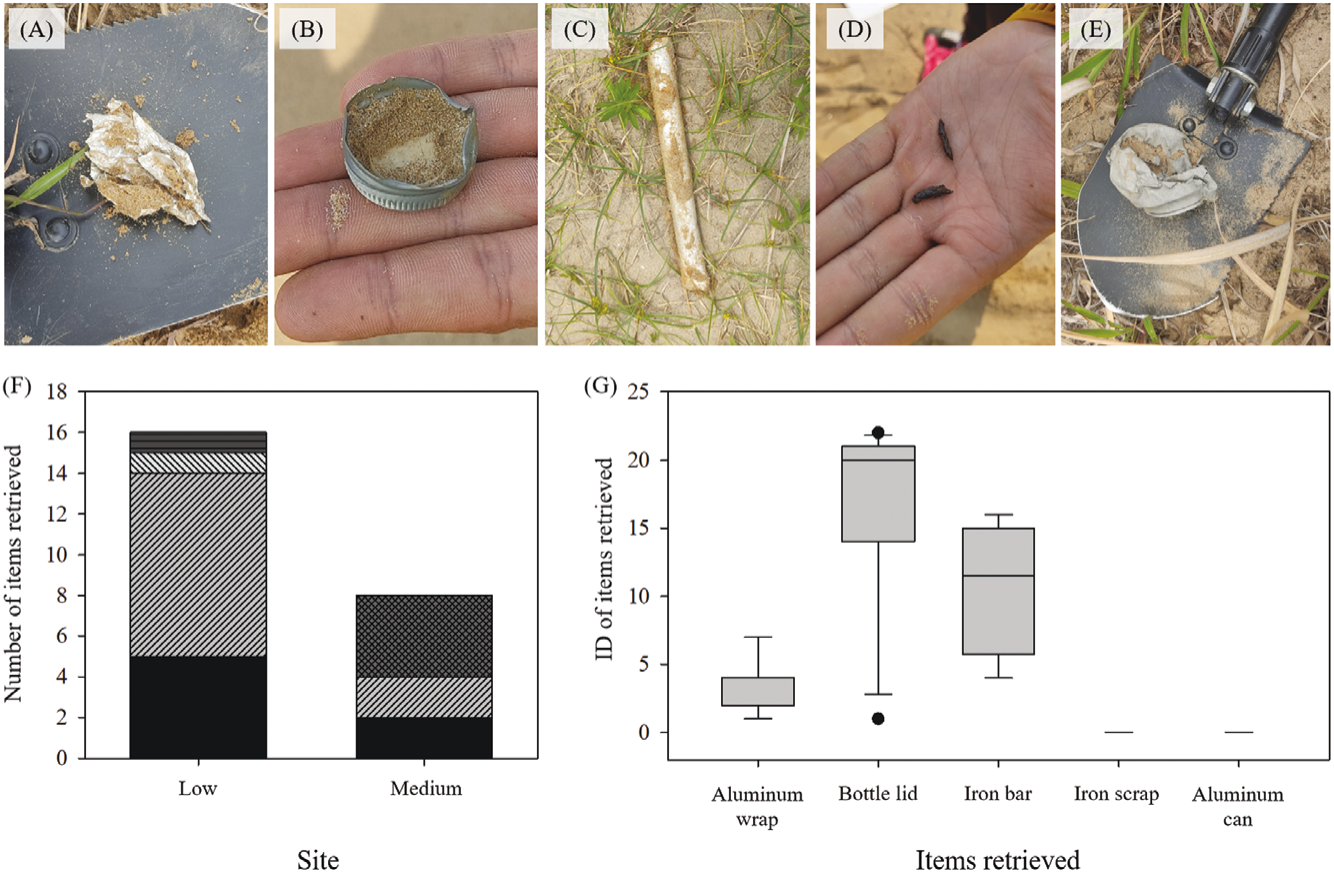
Pre-existing items and associated ID^a^ values detected from low and medium vegetation sites in Taean Sinduri Coastal Dune, South Korea. Low vegetation site consisted of grass up to 20 cm in height and vegetation cover up to 30%, whereas medium vegetation site consisted of grass up to 50 cm in height and vegetation cover up to 70%. Note that only single item with ID value of 0 was retrieved for iron scrap and aluminum can. Aluminum wrap (A), bottle lid (B), iron bar (C), iron scrap (D), and aluminum can (E). Number of items retrieved from 2 sites (F) and ID of items retrieved (G). ^a^ID is a parameter evaluated and presented by a metal detector (Vanquish 540—Minelab Electronics, South Australia, Australia) when sensing aluminum-tagged models.

From screening of 2 vegetation sites for pre-existing metal items, a total of 24 items were detected using a metal detector; 16 and 8 items were detected from low and medium vegetation sites, respectively (Fig. 2). Detected items were aluminum wraps, bottle lids, iron bars, iron scraps, and aluminum cans (Fig. 2A–F). ID value varied among different items. Bottle lids showed the highest IDs among the items, mostly ranging from 14 to 22, followed by iron bar with ID range from 4 to 16 (Fig. 2G). On the other hand, aluminum wraps showed IDs ranging from 1 to 7.

**Fig. 3.**
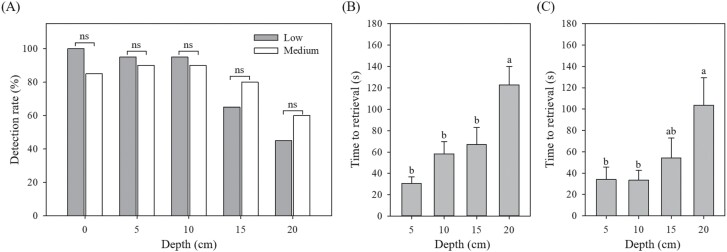
Detection rates of aluminum-tagged models (large, 16-layered) hidden at varying depth in low and medium vegetation sites in Taean Sinduri Coastal Dune, South Korea (A) and time to retrieve the hidden aluminum-tagged models after the metal detector sensed the models at varying depths in low vegetation site (B) and medium vegetation site (C). Note that time to retrieval for 0 cm depth was not included in the data as aluminum-tagged models were immediately noticed while marking their positions. Different letters from each graph indicate significant difference among depths, while ns indicates nonsignificant difference (ANOVA followed by Tukey’s HSD: *P *< 0.05).

Overall, the detection efficacy of the metal detector system was significantly affected by the depth at which aluminum-tagged model was buried and hidden. First, detection rate of metal detection system in general decreased with an increase in depth (Fig. 3A). For both sites, a minimum detection rate of 85% was observed for aluminum tags hidden at depths ≤10 cm below the soil, whereas the detection rates at 20 cm decreased to 45% and 60% for low and medium vegetation sites, respectively. A generalized linear model showed that both depth (χ^2^* = *28.79, *df *= 1,196, *P *< 0.0001) and interaction term between depth and site (χ^2^* = *54.45, *df *= 1,196, *P *= 0.02) had significant effects on detection rate, whereas the effect of site was not significant (χ^2^* = *0.66, *df *= 1,196, *P *= 0.42). When detection rates were compared between the 2 sites for each depth, no significant difference was found (*P > *0.05) (Fig. 3A).

Similarly, depth was a significant factor affecting the time to retrieval of the aluminum-tagged model (Fig. 3B,C). A generalized linear model demonstrated that depth had a significant effect on the time to retrieval (χ^2^* = *60.64, *df *= 1,155, *P *< 0.0001), whereas effects of site (χ^2^* = *1.14, *df *= 1,155, *P *= 0.29) and interaction between depth and site (χ^2^* = *0.19, *df *= 1,155, *P *= 0.66) were not significant. From both sites, time to retrieve aluminum-tagged models at 20 cm depth was significantly longer than those at depths of either 5 and 10 cm for low vegetation site (*F = *6.68, *df *= 3,54, *P *< 0.001) and medium vegetation site (*F = *4.64, *df *= 3,60, *P *< 0.01) (Fig. 3B,C).

### Effect of Aluminum Tagging on Dung Beetles

**Fig. 4.**
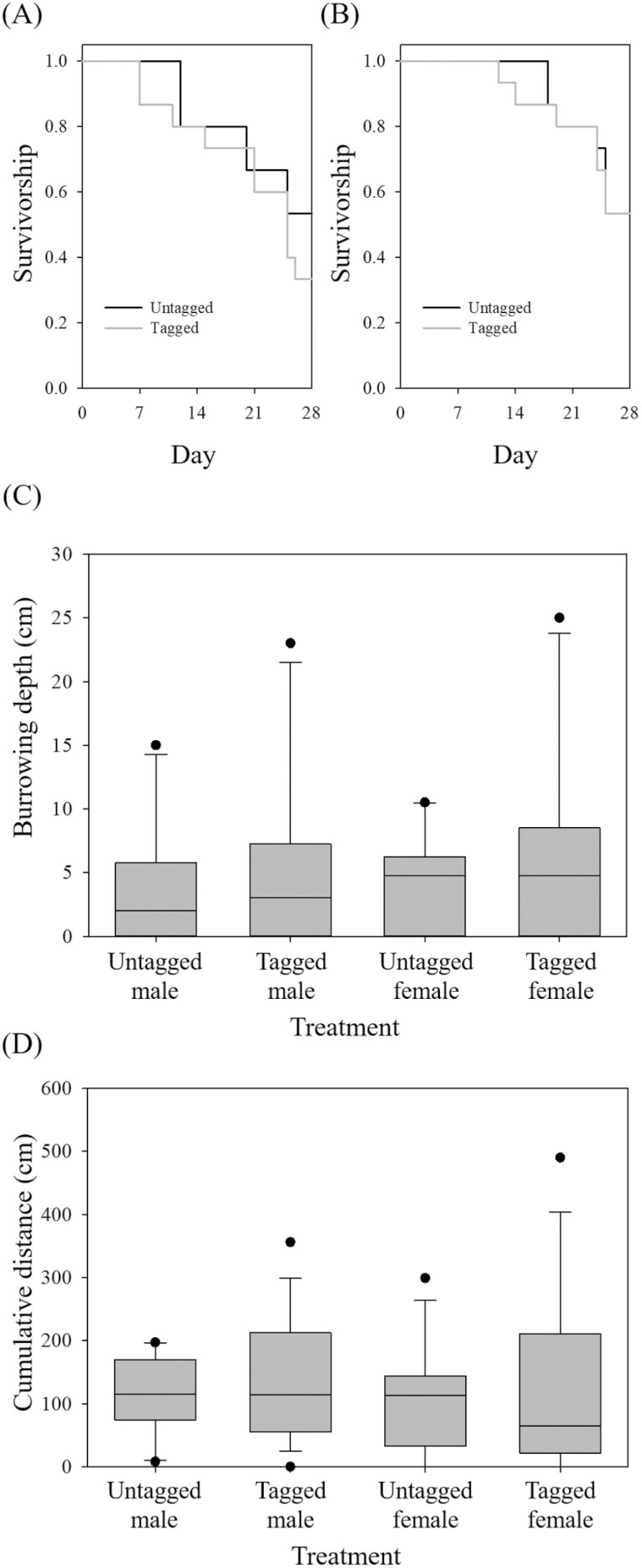
Effect of aluminum tagging (large, 16-layered) on *Copris ochus* adults, including survivorship of *C. ochus* male (A) and female (B); burrowing depth of *C. ochus* male and female (C); and cumulative horizontal walking distance of *C. ochus* male and female (D) over 30 min.

Application of aluminum tags did not have any significant effects on survivorship, burrowing depth, and horizontal movement on the grassland tested in this study (Fig. 4). First, the survivorship of *C. ochus* adults was not significantly different between untagged and aluminum-tagged individuals (male: χ^2^* = *0.96, *P = *0.33; female: χ^2^* = *0.01, *P = *0.91) (Fig. 4A,B). Over the 28 days of the experiment, 8 untagged males and 5 aluminum-tagged males survived, while 8 females survived in both untagged and aluminum-tagged groups.

Second, the generalized linear model showed that none of the aluminum tagging (χ^2^* *= 1.24, *df = *1,36, *P *= 0.27), sex (χ^2^* *= 0.29, *df = *1,36, *P *= 0.59), and interaction between the 2 factors (χ^2^* *= 0.04, *df = *1,36, *P *= 0.84) had a significant effect on the burrowing depth of *C. ochus* adults in the soil (Fig. 4C). Depths under soil where *C. ochus* were detected were 3.5 ± 1.5 and 5.2 ± 2.2 cm (mean ± SE) for untagged and aluminum-tagged males, respectively. For females, depths were 4.1 ± 1.2 and 6.5 ± 2.4 cm for untagged and aluminum-tagged individuals, respectively. In both untagged and aluminum-tagged groups, >80% of individuals were detected within 10 cm under soil, whereas 2 aluminum-tagged individuals were detected >20 cm under soil.

Finally, the mixed model showed that none of the aluminum tagging (*F *= 1.12, *df = *1,16, *P *= 0.30), sex (*F *= 0.00, *df = *1,16, *P *= 0.98), and interaction between the 2 factors (*F *= 0.00, *df = *1,16, *P *= 0.98) had a significant effect on the horizontal walking distance of *C. ochus* adults. Untagged and aluminum-tagged males walked 110.3 ± 16.7 and 134.8 ± 25.2 cm with maximum distance of 197 and 356 cm observed, respectively. For the females, the untagged and aluminum-tagged individuals walked 110.2 ± 21.9 and 135.9 ± 37.6 cm, respectively with a maximum distance of 299 and 490 cm observed, respectively (Fig. 4D).

## Discussion

*Copris ochus* is a paracoprid dung beetle distributed in East Asia including South Korea, China, and Japan. Especially in South Korea, this beetle is one of the few remaining species after the great decline in abundance and diversity of dung beetles (Bang et al. 2004). Currently, its phenology, overwintering, and ecological role have been studied (Bang et al. 2004, 2005, 2008). On the other hand, little is known about its behavior including foraging and dispersal patterns during pre-reproductive stage prior to nesting stage, which would play an important role in understanding the ecology of this species and formulating an effective species conservation strategy. Mark and recapture in association with dung-baited traps has served as one of the most frequently used methods to study the distribution and behavior of dung beetles (Cultid-Medina et al. 2015, Silva and Hernández 2015, Barretto et al. 2023). However, this approach may not be as effective without existing knowledge of their dispersal capacity or the reach of the lure (Miller et al. 2015, Kirkpatrick et al. 2019). Instead, we developed a metal detector system for dung beetles that could be used to locate target species without affecting its on-site distribution and behavior.

The metal detector system developed in our study resulted in a maximum detection distance of 17 cm below soil when varying specifications of aluminum tags were tested, where increase in detection distance was observed with the increase in size and thickness of the tags. Also, in field conditions consisting of 2 different sites varying with vegetation height and cover, large, 16-layered aluminum tags attached to *C. ochus* dummy models could reliably be detected with detection rates ≥85% up to 10 cm below soil and 45%–60% at 20 cm below the soil. Both maximum detection depths measured in semi-field and field conditions were greater than detection depths of metal detectors (Piper and Compton 2002) and harmonic radars (O’Neal et al. 2004) from previous studies, suggesting the potential of our metal detector system for tracking soil-dwelling dung beetles.

Our study also demonstrated that detection efficacy of the metal detector system was mainly affected by the depth at which the aluminum-tagged models were buried and hidden. First, detection efficacy of metal detector system was mainly affected by the depth of target buried regardless of the vegetation type. As expected, while the detection rate generally decreased with depth, the time to retrieval showed an increase in both low and medium vegetation sites. Nevertheless, the method allowed retrieval of all sensed aluminum-tagged models within 5 min at all depths, suggesting the viability of the metal detector system in both environments. Furthermore, the effect of orientation of the aluminum-tagged model on the detection efficacy was almost negligible unless the aluminum-tagged model was placed completely perpendicular to the ground. Generally, the variation in detection rates was within 30% when the orientations were 0°, 45°, 135°, and 180°. These results demonstrate the robustness of the metal detector system for detecting target species applicable in diverse field conditions.

The metal detector system used in this study allowed us to detect only target aluminum tags of desired ID. When we conducted field screening of metal items in coastal dune sites, the most frequently found items were artificial objects including bottle lids and aluminum wraps, which had ID range of 14–22 and 1–7, respectively. In such an environment, large 16-layered aluminum tags used in this study, which yielded the most frequently detected ID of 9, could be detected without interference of potential false signals originating from nontarget metal objects. Thus, screening of study sites preceding the release of metal-tagged insects could help determine specifications and ID of tags to be used for experiments, which is important for an accurate and efficient application of the method.

Overall, aluminum tagging did not have a significant effect on the survivorship, burrowing depth, and horizontal movement of *C. ochus*. However, we did not evaluate the effect of aluminum tagging on different aspects of the behaviors of *C. ochus* including the flight or mating, which need to be further assessed in future studies. In preliminary experiments, we opted to identify conditions where the insects could initiate flights via various approaches including video recording over 10 days, exposure to light source, starvation, and throwing them in the air, none of which was successful. Indeed, flight was observed from only 20% of *C. ochus* when their flight capacity was evaluated using flight mill experiment in the laboratory (unpublished data). Especially, due to the importance of flight in dispersal and foraging behavior of *C. ochus* (Silva and Hernández 2015), the effect of aluminum tagging on the flight needs to be evaluated. Previous studies suggested that acceptable tag-to-body weight ratio may vary for different species, especially for flight. For example, Kim et al. (2016) reported that attachment of 3 mg tag on 3 different insect species, *Riptortus pedestris* (Hemiptera: Alydidae) (41 mg), *A. mellifera* (100 mg), and *Ricania* sp. (28 mg), had varying effects on flight. Whereas flight behavior of *R. pedestris* was not affected by tag attachment, takeoff propensity decreased by 27% for *A. mellifera*. Also, both takeoff propensity and flight capacity were adversely affected for *Ricania* sp. Likewise, a more thorough understanding of the effect of tagging on behaviors of *C. ochus* will allow us a more accurate observation of the insect in the field.

Despite the potential of the metal detector system for studying *C. ochus* demonstrated in this study, the applicability of the method may vary depending on ecological traits of the target species. In our study, the maximum detectable distance evaluated for varying tag specifications was 17 cm in semi-field conditions, and aluminum-tagged models buried up to 20 cm below soil could be detected with success rate of 45%–60% in the field. However, *C. ochus* adults build reproductive burrows where they breed and care for their offspring over the winter (Bang et al. 2008). These reproductive burrows are known to be located at a range of 22–35 cm below soil (Bang et al. 2004). Therefore, the metal detection system in this study might not be readily applicable when detecting *C. ochus* in reproductive and overwintering states. However, *Copris* species are reported to build feeding burrows under or near dung pads prior to reproduction that are shallower than reproductive burrows (Blume and Aga 1976, Doube 1990, Price and May 2009). Indeed, in our laboratory experiment, >80% of *C. ochus* adults were distributed within 10 cm below soil when placed overnight in an experimental arena filled with soil. Therefore, our method can be applied to track *C. ochus* from their spring emergence until their reproductive stage known as pre-reproductive phase during which adults actively disperse and forage and is functionally important for the ecosystem.

## Supplementary Material

ieae067_suppl_Supplementary_Material
