# Correction to: Development of underground detection system using a metal detector and aluminum tag for, Copris ochus (Coleoptera: Scarabaeidae)

**DOI:** 10.1093/jisesa/ieae081

**Published:** 2024-07-27

This is a **correction** to:

Jung-Wook Kho, Young-Joong Kim, Hwang Kim, Sun Hee Hong, Young Su Lee, Jong-Seok Park, Doo-Hyung Lee, Development of underground detection system using a metal detector and aluminum tag for, *Copris ochus* (Coleoptera: Scarabaeidae), *Journal of Insect Science*, Volume 24, Issue 3, May 2024, 27, https://doi.org/10.1093/jisesa/ieae067

The following change has been made to the original publication of the article. The title has been edited to read: “Development of underground detection system using a metal detector and aluminum tag for Copris ochus (Coleoptera: Scarabaeidae)” instead of: “Development of underground detection system using a metal detector and aluminum tag for, Copris ochus (Coleoptera: Scarabaeidae)”.

