# Supplementary material for: Development of underground detection system using a metal detector and aluminum tag for Copris ochus (Coleoptera: Scarabaeidae)
Source: J Insect Sci. 2024 Jun 24;24(3):27. doi: 10.1093/jisesa/ieae067 (PMC11195468; doi:10.1093/jisesa/ieae067)

Supplementary Fig. 1 Plastic dummy model of *Copris ochus* applied with aluminum tags (large, 16-layered) (A), aluminium-tagged *C. ochus* male (B) and female (C).


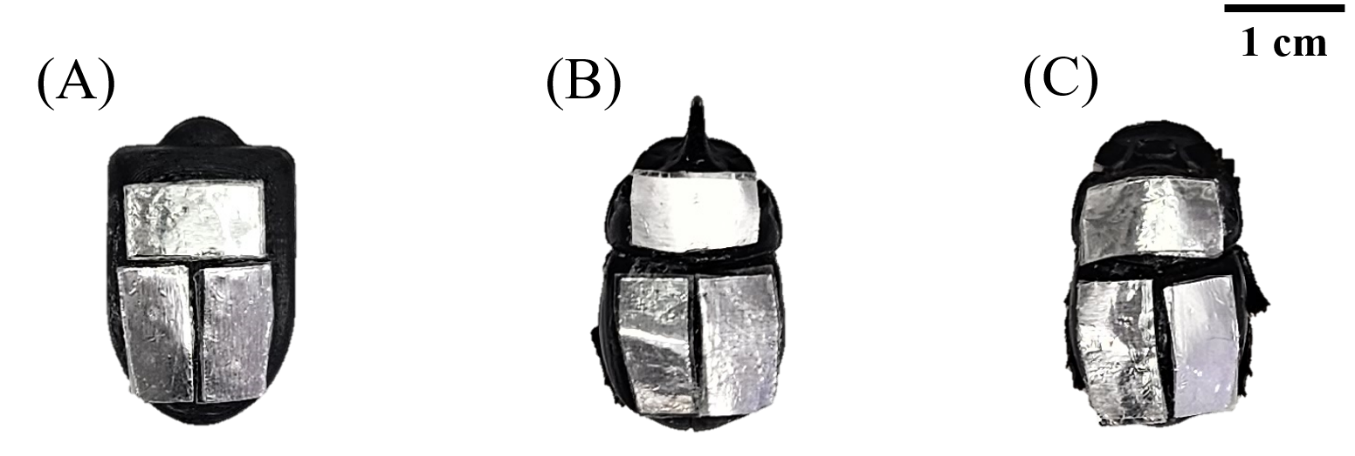

Supplement: ieae067_suppl_Supplementary_Material [file ieae067_suppl_supplementary_material.docx]
